# Virtual teaching of preventive cardiology: A student experience

**DOI:** 10.1016/j.ajpc.2020.100107

**Published:** 2020-10-21

**Authors:** Sara Hays, Vikas Mankala, Kevin Paternostro, Deepraj Pawar, Edward Ward, Lauren Raymond

**Affiliations:** Division of Cardiovascular Medicine, Oregon Health & Science University, Portland, OR, United States; Division of Cardiovascular Medicine, Oregon Health & Science University, Portland, OR, United States; Division of Cardiovascular Medicine, Oregon Health & Science University, Portland, OR, United States; Division of Cardiovascular Medicine, Oregon Health & Science University, Portland, OR, United States; Division of Cardiovascular Medicine, Oregon Health & Science University, Portland, OR, United States; Division of Cardiovascular Medicine, Oregon Health & Science University, Portland, OR, United States

**Keywords:** Preventive cardiology, Medical education, Virtual education, Telehealth

## Abstract

Preventive cardiology is a subspecialty of Internal Medicine focused on reducing atherosclerotic cardiovascular disease (ASCVD) risk through medical management of known risk factors and identification of genetic predispositions. Oregon Health & Science University (OHSU) provides a preventive cardiology course designed to engage students in targeted, multidisciplinary care to address the increasing ASCVD prevalence. Following the onset of the COVID-19 pandemic, OHSU transitioned this course to a virtual platform to allow students to continue their medical education. Course adaptations include utilization of video-conferencing platforms and cloud-based storage websites, allowing students access to didactic materials, instructional sessions, and engage with patients in a telehealth setting. As the first cohort of students to complete this course, we share our experience with the virtual platform, including the virtual course structure, student role, and benefits and limitations of this model. Through our experience, we have found that adaptation to a virtual platform provides a feasible and effective means through which to continue preventive cardiology education during the COVID-19 pandemic.

## 1 Introduction

In 2016, the prevalence of cardiovascular disease in the United States was 48%, or 121.5 million people, in adults greater than age 20 [1]. Cardiovascular disease is also the leading cause of death globally [2]. Preventive cardiology is a subspecialty focused on reducing the risk of heart disease and major adverse cardiac events, such as myocardial infarction or stroke, in both patients who do and do not have pre-existing heart disease [3]. Given the prevalence of heart disease both in the United States and worldwide, continued medical student instruction in preventive cardiology is crucial, even as the COVID-19 pandemic forces a shift towards virtual medical education.

The field of preventive cardiology emerged decades ago, as healthcare providers learned more about the modifiable risk factors of heart disease. The subspecialty specifically involves the management of conditions such as hypertension, diabetes, dyslipidemia, and obesity, along with assessment of lifestyle factors, genetics, and high-risk biomarkers [4]. The primary aim is to assess the risk of cardiovascular disease and cardiovascular events in each patient, many of whom may have pre-existing cardiovascular conditions or genetic predispositions, then employ management strategies to mitigate risk. It is a multidisciplinary field, including teams of cardiologists, endocrinologists, nurse practitioners, dietitians, and naturopaths. While there are no established competencies for preventive cardiology training at this time, one-year fellowship programs are beginning to emerge [5]. It has even been suggested that preventive cardiology fellowships be offered to internists, endocrinologists, and family medicine physicians, reinforcing the importance and breadth of this field [4]. Oregon Health & Science University (OHSU) currently offers a 2-week preventive cardiology elective to medical students and has continued to offer this course during the COVID-19 pandemic via an adapted virtual platform.

As one of the first cohorts of medical students to take this elective virtually at OHSU, we aim to discuss the teaching modalities of the traditional clinical elective, as well as its adaptation to a virtual platform during the COVID-19 pandemic. Developing a virtual course has been an adaptive process with the goal of optimizing the structure of the course, the role of the instructors and students, and the technologies used. By providing information about the curriculum, as well as the medical student experience, we hope to show that this is a feasible and effective alternative to traditional in-person clinical training.

## 2 Teaching modalities

The framework for teaching preventive cardiology during medical education has traditionally involved several different modalities for students to learn skills and translate them in the clinical setting [6]. Curricula commonly utilize a combination of in-person didactic lecture and clinical work, designed to address competencies outlined by the Accreditation Council of Graduate Medical Education (ACGME) or similar governing bodies [7].

### 2.1 Didactic lectures

In medical education, didactic lectures are meant to supplement the clinical experience. According to Sackett et al., didactic lecture in conjunction with clinical expertise and exploration of evidence-based practice is necessary for effective medical training [8]. Traditionally, such lectures involve an instructor presenting content to students who are passive listeners. Many institutions utilize this teaching style in part or in whole for teaching cardiovascular health and management. While the teacher-centered method has traditionally been utilized in the modern education system, more institutions have incorporated active learning practices within their didactic education. This approach has been validated in teaching science and physiology [9], and has had strong satisfaction amongst medical students [10]. Active learning within a preventive cardiology curriculum can take the form of student-led journal clubs, small group discussion, and case studies, amongst other techniques. Some features of the Mayo Clinic Preventive Cardiology fellowship include clinical conferences, seminars, small discussion groups, and journal clubs, which are integral in the training of the fellows at that program [10].

Another consideration for the didactic lecture modality is the medium of content delivery, notably in-person instruction versus virtual instruction. Traditional instruction of preventive cardiology at our institution and others has favored the in-person classroom approach. However, with the advance of technology and contexts now requiring an asynchronous learning environment, such as the COVID-19 pandemic, the use of a virtual learning environment to teach preventive cardiology is a consideration.

### 2.2 Preventive cardiology clinics

In-person preventive cardiology clinical training constitutes the core of the clinical education experience. Under the supervision of a health practitioner, medical students and residents have the opportunity to learn and practice preventive cardiology care, typically in a teaching hospital. This can take the form of in-person observation of the practitioner-patient interaction, patient interview, completion of a history and physical, electronic health record documentation, laboratory test and imaging interpretation, and patient presentation. Two institutions that incorporate clinical experiences include The University of Nevada Reno School of Medicine (UNR) and The David Geffen School of Medicine at the University of California in Los Angeles (UCLA). UNR currently offers a 2–4 week vascular medicine and preventive cardiology elective for students [11]. Other programs may not have a dedicated preventive cardiology elective, instead educating learners on components of preventive cardiology through a general cardiology consult elective. Medical students at UCLA have the opportunity to learn preventive cardiology from fellows at Cedars-Sinai through a general cardiology consult rotation [12].

### 2.3 Multimodal approach

At OHSU, instructors utilize a multimodal approach of didactic lecture and clinical skills training to teach preventive cardiology to medical students. Outlined as follows is the course structure of the preventive cardiology elective at OHSU before COVID-19. Prior to the start of the course, students drafted a learning plan with individualized goals. A clinical schedule was designed based on a student’s goals and desired depth of involvement. Over the span of two weeks students had the opportunity to participate in preventive cardiology clinics, working with several different providers, including cardiologists, endocrinologists, nurse practitioners, and dietitians. In the clinic, students could conduct patient interviews, present cases to the care team, and complete a full history and physical as desired. Didactic lectures were held in person with faculty and targeted to learning needs, with topics including lipid metabolism, diet and obesity, cardiovascular health and management, and pharmacology. Supplemental information to didactic lectures was available to students in the form of relevant readings and papers, delivered through a cloud sharing service. Course enrollment was usually limited to 1–2 students, and there was little student collaboration given this course structure.

## 3 Virtual course structure & student role

In the era of telehealth and teleconferencing, the structure of the preventive cardiology elective at OHSU has shifted to include daily didactic lectures online, weekly virtual clinic meetings, and near-daily clinical work through telemedicine. We started our two-week elective with an orientation to both the preventive cardiology clinical setting as well as the new virtual telehealth platform. Following the orientation, we began with didactic lectures on the basics of lipoprotein metabolism, genetic dyslipidemia, low-density lipoprotein apheresis, and risk assessment tools for cardiovascular disease to deepen our understanding of preventive cardiology as a subspecialty before seeing our first patients. Throughout the elective, we also learned about obesity management, diastolic dysfunction, preventive measures to combat congestive heart failure, dietary and supplemental interventions, cardiac rehabilitation, pharmacology and the role of the clinical pharmacist, the role of naturopathy in preventive cardiology, and ongoing research within the field. We were given access to lecture materials before our didactic sessions and encouraged to come prepared with questions in order to maximally engage in these sessions.

Supplementing this didactic content, the bulk of the elective focused on the clinical practice of preventive cardiology. As students, we participated in telehealth-based patient encounters with a number of different providers, including physicians, nurse practitioners, and a dietitian. Prior to the start of each clinical session, we would read about the patients for the day and self-assign patients to present to the provider before the start of the encounter. We would then discuss the patient’s history with the provider and receive real-time feedback about our presentations, including the most salient clinical details and additional information to consider obtaining from the patient during the upcoming encounter. Prior to student inclusion in the encounter, the provider discussed the details of virtual medical education with the patient and obtained consent. Once included in the visit, we were encouraged to ask clarifying questions to the patients throughout the encounter to build upon the information we had discovered during chart review. We were also asked to develop treatment plans for the patient based upon didactic content and patient goals. Although we were unable to physically examine the patient, the time spent studying course content and applying these concepts to patients was invaluable in solidifying our understanding of the unique patient population of preventive cardiology.

## 4 Technology

Since the onset of the COVID-19 pandemic, many medical schools have adapted their present technologies for remote learning and continue to be creative in their use of newer technologies [13]. As discussed by Newman and Lattouf, there are several means through which institutions can transition clinical education to a virtual platform [14]. Our preventive cardiology program made use of many resources to effectively transition their curriculum to an online platform, including the following:

Conferencing platforms: Video-conferencing platforms, such as Zoom, Skype, or in our case, Cisco WebEx, can be used to host classes, clinical sessions, and meetings online. Our course featured both didactic and clinical sessions with department faculty, all held over Cisco WebEx. During clinical sessions, faculty would share their screens to allow students to observe how they interacted with patients and how they navigate the patient chart. Though students and patients were hosted on independent conferencing platforms, their connection through the faculty computer enabled verbal communication, allowing students to conduct interviews with patients.

Storage platforms: Cloud-based storage sites designed to house educational resources, including recordings, publications, and text, create a convenient way to share educational information with students. Examples of commonly used software include Box, DropBox, and Zotero. Usage of these platforms was imperative to our online curriculum, which relied on independent reading of provided resources prior to didactic sessions.

Third party educational websites: Resources such as web-based informational subscriptions and question banks aid in students’ understanding of the course content. Our institution maintained a partnership with OnlineMedEd throughout the duration of our course. This allowed us access to a wide array of educational videos, practice cases, and questions, all designed to improve background knowledge and clinical understanding. Similar platforms free to all students regardless of institutional partnership include the AAMCs iCollaborative [15] and MedEdPortal [16].

Inevitably, the application of technology to improve the quality of our virtual courses will change over time. Such improvements will allow for enhanced clinical training within preventive cardiology and similar specialties. This process is currently underway within our own institution, with administration working to allow students access to patient encounters through the same platform as faculty. As students, we are unable to comment on the financial feasibility of these transitions, as the transition occurred on a university level within our institution.

## 5 Pros & CONS OF the virtual learning environment

**Fig. 1:**
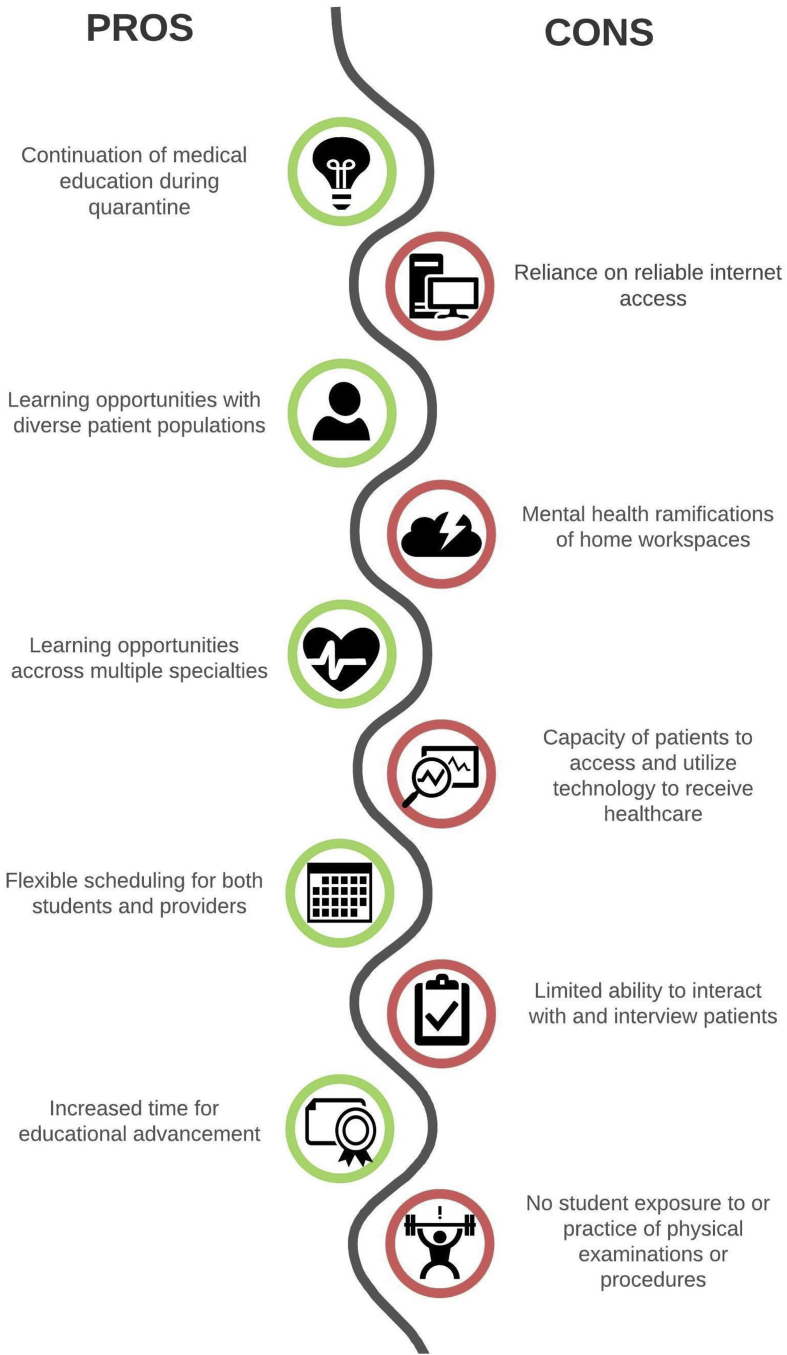
Pros and cons of the virtual learning environment for students, educators, and patients.

Adoption of a virtual platform for preventive cardiology and medical education presents a number of pros and cons (Fig. 1). In defense of the virtual environment, the use of a virtual platform accomplishes the primary educational goal during the COVID-19 pandemic: allowing students to continue their medical education and clinical learning remotely. Additionally, remote learning allows students the ability to interact with patients in a wide variety of communities, including both urban and rural populations, the latter of which is typically more difficult for learners to reach. Furthermore, by connecting online rather than in person, students have the opportunity to interact with providers across multiple specialties, a task which may not have been feasible previously due to space, timing, and commuting limitations. Such connections also allow for more flexible scheduling for both students and providers, which is a substantial benefit within the context of our busy schedules. Lastly, reduction of travel time allows students more time to review materials and prepare for future patient care interactions.

Although there are many benefits of adapting medical education to the virtual platform, there are numerous problems with this method of delivery as well. First and foremost, students must have access to both a home workspace and reliable internet connection. A study from Microsoft in September 2019 found that approximately 162 million people in the United States do not have access to broadband internet [17], and here in Oregon, 92% of white households have broadband internet but only 77% of nonwhite, non-Hispanic households have similar access demonstrating that internet access can vary significantly based on demographics [18]. Furthermore, the transition to virtual platforms in conjunction with office closures has forced many to work from home. Although this might be more convenient than traveling for work, there are mental health ramifications to working from home, including blurred work-home boundaries, feelings of isolation, and increased fatigue [19].

More specific to the practice of medicine, the virtual platform requires patients to have access to adequate technology in order to participate in their own healthcare. This will disproportionately affect patients of lower socioeconomic status and elderly patients. During this elective the utilization of telemedicine was still developing at our institution, resulting in frequent troubleshooting and less time spent in direct patient care. As students, we are also limited in our ability to interact with patients on these new virtual platforms. Before the transition to virtual platforms, students often had the opportunity to interview patients before the provider entered the room. With the current restrictions of our virtual platforms, students are now starting patient encounters with the provider and do not have the opportunity to do independent interviewing. Additionally, since there were no in-person encounters, students could not perform physical examinations or participate in office procedures.

## 6 Conclusion

The field of preventive cardiology is rapidly expanding and medical education must keep pace to allow the next generation of physicians to provide optimum patient care. As cardiovascular disease, diabetes, and obesity remain prominent conditions [1,20,21], it is pertinent to focus medical education on learning to prevent severe comorbidities. The preventive cardiology elective at OHSU has elaborated on treatment modalities discussed during didactic training and provided ample clinical learning opportunities. Instruction throughout the elective has offered invaluable insight into multi-organ system interactions, cost effective strategies for care provision, and the importance of multidisciplinary teams in implementing preventive therapies.

As the COVID-19 pandemic developed, clinical electives shifted to the virtual environment to continue student education, including OHSU’s preventive cardiology elective. This transition provided students with opportunities to build upon didactic knowledge, work with a multidisciplinary team, sharpen clinical reasoning, and hone patient interaction skills on a virtual platform. Upon course completion, all students met university-selected ACGME requirements and reported high levels of satisfaction with the virtual learning environment. As future physicians, it is imperative that we are exposed to preventive medicine and telehealth practices so we are better equipped to care for our future patients. Ultimately, our course demonstrates that virtual teaching is an effective, student-supported, and logistically feasible strategy for delivering a preventive cardiology curriculum.

## Financial disclosure

The authors have no financial relationships relevant to this article to disclose.

## Conflicts of interest

The authors declare that they have no known competing financial interests or personal relationships that could have appeared to influence the work reported in this paper.
